# SysML Models for Discrete Event Logistics Systems

**DOI:** 10.6028/jres.125.023

**Published:** 2020-07-29

**Authors:** Timothy Sprock, Conrad Bock

**Affiliations:** 1 National Institute of Standards and Technology, Gaithersburg, MD 20899, USA

**Keywords:** Model-based Systems Engineering, Production Systems, Smart Manufacturing, SysML

## 1 Summary

System models and model-based engineering methods have the promise of transforming the way that industrial engineers interact with production and logistics systems. Model-based methods play a role in improving communication between stakeholders, interoperability between systems, automated access to consistent analysis models, and multi-disciplinary design methods for complex systems. However, there remains a need for a foundation for modeling these kinds of systems – a foundation that tailors methods and tools developed in other engineering domains to the unique concepts and semantics of production and logistics. This foundation is the topic of these models.

This repository contains model libraries for modeling discrete event logistics systems (DELS), an abstraction that covers manufacturing plants, material handling and transportation systems, warehouses, supply chains, etc. The DELS abstraction was created by identifying and modeling commonalities across the kinds of systems that industrial engineers typically encounter, and analysis models they use to analyze those systems. It extends well-known product, process, and resource (PPR) ontologies to incorporate a library of operational control model components, and is connected to Commodity Flow Network (CFN), modeling networks, flow networks, and process networks. The relationship between DELS and CFN formally links system models to abstractions used to create analysis models, such as discrete event simulation.

This repository is the first public release of models and documentation capturing many years of refinement and application by the authors. As a first release, the goal is to solicit additional use cases and feedback from the community to improve the models and make them the foundation for the model-based industrial and systems engineering community.

The models are implemented in the Systems Modeling Language (SysML) [1] developed using MagicDraw^1^1Certain commercial software is identifed in this paper to specify the models adequately. Such identifcation is not intended to imply recommendation or endorsement by the National Institute of Standards and Technology, nor is it intended to imply that the software identified is necessarily the best available for the purpose. 18.5 by No Magic. The models are documented in NISTIR 8262 [2].

**2:** Software Specifications

**NIST Operating Unit**	Engineering Laboratory, Systems Integration Division, Systems Engineering Group
**Category**	Systems engineering model
**Targeted Users**	Industrial and Systems Engineering Practitioners and Researchers.
**Operating Systems**	N/A
**Programming Language**	This software data item is created using SysML 1.4 and stored as Extensible Markup Language (XML) Model Interchange (XMI) models and Hypertext Markup Lan- guage (HTML) reports. MagicDraw 18.5 or later with SysML plugin is required to edit the model (also provided in proprietary mdzip format). The HTML documentation is provided for readers without the MagicDraw software.
**Inputs/Outputs**	N/A
**Documentation**	NISTIR 8262: https://doi.org/10.6028/NIST.IR.8262 Source models: https://github.com/usnistgov/DiscreteEventLogisticsSystems
**Accessibility**	N/A
**Disclaimer**	https://www.nist.gov/director/licensing

## 3 Methods

### 3.1 Implementation Tool

The SysML model was implemented using a proprietary graphical modeling tool called MagicDraw. MagicDraw supports the Unifed Modeling Language (UML) [3] and SysML [1] among other graphic modeling languages. The SysML models can be exported in OMG XML Metadata Interchange (XMI) [4] fle format and Hypertext Markup Language (HTML) reports.

### 3.2 SysML Diagram Types

The following SysML diagrams are used for communicating block relationships and architecture:

• Package Defnition Diagram (PKG) – used to capture package containment and relationships;
• Block Defnition Diagram (BDD) – used for capturing blocks, interfaces, and their relationships;
• Class Defnition Diagram (CD) – used for capturing software classes, interfaces, and relationships;
• Activity Diagram (ACT) – used to capture behaviors, sequence of execution, and fows between executions;
• Internal Block Diagram (IBD) – used for capturing the internal composition of a single block.

### 3.3 Model Structure

• Commodity Flow Network
  – Networks
  – Flow Networks
  – Process Networks
• Discrete Event Logistics Systems
  – Context
  – Plant
  _*_ Product
    _*_ Process
    _*_ Resource
    _*_ Facility
  – Control
